# Performance of ^18^F-fluorodesoxyglucose positron-emission tomography combined with low-dose computed tomography for cancer screening in patients with unprovoked venous thromboembolism

**DOI:** 10.1371/journal.pone.0178849

**Published:** 2017-06-01

**Authors:** Philippe Robin, Pierre-Yves Le Roux, Karine Lacut, Benjamin Planquette, Nathalie Prévot-Bitot, Christian Lavigne, Jean Pastre, Adel Merah, Grégoire Le Gal, Pierre-Yves Salaun

**Affiliations:** 1 Service de Médecine Nucléaire, EA 3878 (GETBO) IFR 148, Centre Hospitalo-Universitaire de Brest, Université de Bretagne Occidentale, Brest, France; 2 Département de Médecine Interne et Pneumologie, EA 3878, CIC INSERM 1412, Centre Hospitalo-Universitaire de Brest, Université de Bretagne Occidentale, Brest, France; 3 Service de Pneumologie, Hôpital Européen Georges Pompidou, AP-HP; Université Paris Descartes, Sorbonne Paris Cité, INSERM UMR-S 1140, Paris, France; 4 Service de Médecine Nucléaire, Inserm U1059, Centre Hospitalo-Universitaire de Saint-Etienne, Université Jean Monnet, Saint-Etienne, France; 5 Département de Médecine Interne et Maladies Vasculaires, Centre Hospitalo-Universitaire d’Angers, Angers, France; 6 Service de médecine vasculaire et thérapeutique, Inserm CIC 1408, Centre Hospitalo-Universitaire de Saint-Etienne, Saint- Etienne, France; 7 Department of Medicine, Ottawa Hospital Research Institute at the University of Ottawa, Ottawa, Canada; North Shore Long Island Jewish Health System, UNITED STATES

## Abstract

**Purpose:**

Small series have suggested that Fluorodesoxyglucose Positron-Emission-Tomography with Computed-Tomography (FDG-PET/CT) is feasible to screen for cancer in patients with unprovoked venous thromboembolism (VTE), but without validation in a large population. The aim was to assess diagnostic accuracy indices of FDG-PET/CT for occult cancer diagnosis in patients with unprovoked VTE.

**Materials and methods:**

We analysed patients from the FDG-PET/CT group of a randomized trial that compared a screening strategy based on FDG-PET/CT with a limited screening strategy for occult malignancy detection in patients with unprovoked VTE. FDG-PET/CT was interpreted as positive for cancer, as negative or as equivocal. Patients were considered as having cancer on the basis of screening results, or of any test performed during a two-years follow-up period. We ran two sets of analysis, considering patients with equivocal FDG-PET/CT as positive, then as negative for malignancy.

**Results:**

Between March 2009, and August 2012, 172 patients were included. FDG-PET/CT was interpreted as positive for malignancy in 10 patients (5.8%), as equivocal in 23 patients (13.4%) and as negative in 139 patients (80.8%). Malignancy was diagnosed in 7/10 (70.0%), 2/23 (8.7%) and 1/139 (0.7%) patients, respectively. Grouping positive and equivocal results, sensitivity and specificity were 90% (95%CI 60% to 98%) and 85% (95%CI 79% to 90%), respectively. Grouping negative and equivocal results, sensitivity and specificity were 70% (95%CI 40% to 89%) and 98% (95%CI 95% to 99%), respectively.

**Conclusion:**

FDG-PET/CT showed good accuracy for occult cancer screening in patients with unprovoked VTE. Remaining challenges include the need to define specific interpretation criteria in this dedicated population.

## Introduction

^18^F-Fluorodesoxyglucose Positron-Emission Tomography combined with low-dose Computed Tomography (FDG-PET/CT) is routinely used for the diagnosis, staging and restaging of various malignancies [1,2]. However, much less is known on the performance of FDG-PET/CT for occult malignancy screening in selected subgroups of patients. Screening for occult malignancy may allow for earlier detection and treatment of cancer, thus improving the prognosis, and is therefore appealing for patients known to be at increased risk of cancer.

Venous thromboembolism (VTE), which encompasses deep-vein thrombosis and pulmonary embolism, can occur as the first manifestation of an underlying occult malignancy [3]. Previous studies reported that the incidence of undiagnosed cancer is 6% to 15% in the year after an unprovoked VTE episode (i.e., VTE not provoked by a major inherited or acquired risk factor including surgery, trauma or fracture during the three months prior to the venous thromboembolic event; known antiphospholipid antibody syndrome, known deficiency in antithrombin, protein C or protein S) [4–10]. Screening for occult malignancy at the time of VTE has been advocated. Different extensive occult cancer screening strategies have been proposed [11–14]. Because all types and locations of cancer may be found in patients with VTE, many investigations had to be performed for screening and as a result an extensive screening strategy is required, which is expensive, invasive and time-consuming [12]. Clear guidelines for the investigation of occult malignancy after unprovoked VTE are not yet available.

FDG-PET/CT has the advantage of providing non-invasive whole body imaging. Small case series have suggested that FDG-PET/CT is a feasible and sensitive test to screen for cancer in patients with unprovoked VTE [15–17]. More recently, we reported the results of a randomized trial comparing a limited screening strategy (medical history, physical examination, routine laboratory and age/gender recommended screening tests) to a strategy combining the limited screening plus a FDG-PET/CT [18]. We found the use of FDG-PET/CT not to be associated with a significantly higher rate of cancer diagnosis at initial screening. However, the incidence of subsequent cancer diagnosis over a two-year follow-up period was significantly lower in patients randomized in the limited plus FDG-PET/CT strategy.

As an ancillary analysis, we aimed at evaluating the diagnostic accuracy indices of FDG-PET/CT for the diagnosis of occult malignancy in patients included in the limited plus FDG-PET/CT strategy arm.

## Materials and methods

### Study population

We analysed patients from the FDG-PET/CT group of an open label, multicenter (four university medical centers in France), randomized study that compared a screening strategy based on FDG-PET/CT with a limited screening strategy for detection of occult malignant disease in patients with unprovoked VTE. Methods have been detailed elsewhere [18].

Briefly, patients aged 18 years or older, diagnosed with unprovoked VTE were invited to participate in the study if they did not present any exclusion criteria: ongoing pregnancy, active malignancy (defined as known malignancy, active and/or treated during the previous five years). Unprovoked VTE was defined as not provoked by a major inherited or acquired risk factor including surgery, trauma or fracture during the three months prior to VTE; known antiphospholipid antibody syndrome, known deficiency in antithrombin, protein C or protein S.

In the limited plus FDG-PET/CT arm, patients underwent medical history, complete physical examination, routine laboratory tests including complete blood count, erythrocyte sedimentation rate or C-reactive protein, transaminases, alkaline phosphatase, calcium, chest X-ray, recommended age- and gender-specific cancer screening tests (i.e. prostate-specific antigen in men over 50 years of age, mammography in women over 50 years of age and Pap-smear in all women). All patients were scheduled for a FDG-PET/CT, to be performed within four weeks of inclusion (see below). In case of positive finding on initial screening, patients were referred for appropriate diagnostic procedures at the discretion of the treating physician. All patients underwent clinical follow-up every 6 months for 24 months. Medical history and physical examination were performed and in case of new symptoms or clinical signs, further testing was ordered. Information on any investigation for suspected malignancy requested by other physicians during follow-up was collected.

The study was conducted in accordance with the ethical principles set forth in the Declaration of Helsinki, Good Clinical Practice, and relevant French regulations regarding ethics and data protection. All patients provided written consent. The protocol and amendments were approved for all study sites by our institutional Ethics committee (Comité de Protection des Personnes Ouest VI, 2008–541).

### FDG-PET/CT acquisition

FDG-PET/CT were performed using Gemini GXLi, Philips in Brest University Hospital; Discovery ST, General Electric in Angers University Hospital; Biograph 6 LSO Pico 3D HI-REZ, Siemens Medical in Saint Etienne University Hospital; Gemini GXL, Philips and Discovery 690, General Electric in Paris. Patients fasted for at least 6 hours before PET acquisitions, and blood glucose had to be less than 7 mmol/L before injection of 3 to 5 MBq/kg of 18F-FDG. Intravenous injection was followed by a period of approximately 60 minutes when the patients remained in a quiet room. Computed tomography was performed from mid-forehead to the feet in normal shallow respiration using a low-dose setting (120 kVp—100 mAs in Brest; 120 kVp—50 mAs in Angers and Paris; 130 kVp—50 mAs in Saint-Etienne). Intravenous iodinated contrast was not administered. Data obtained from the CT-scan were used for attenuation correction of PET data and for fusion with attenuation-corrected PET images. PET data was reconstructed iteratively using the ordered-subset expectation maximization (OSEM) algorithm (2 iterations and 8 subsets in Angers and Brest; 2 iterations and 16 subsets in Paris; 4 iterations and 8 subsets in Saint Etienne).

### FDG-PET/CT interpretation

For the purpose of this analysis, all FDG-PET/CT scans were centrally reinterpreted at the main study site by a board-certified nuclear medicine physician, after the trial was completed, and without knowledge of patient’s clinical information and follow-up status. FDG-PET/CT was interpreted as positive for possible malignancy based on the following criteria: foci of non-physiological FDG uptake, not attributable to the acute venous thromboembolism (i.e. areas of vascular thrombosis or lung infarcts were excluded), with no predetermined cut-off maximum standardized uptake values. FDG-PET/CT was interpreted as negative when only physiological FDG uptake or non-physiological FDG uptake attributable to a typical benign disease (e.g. global homogeneous thyroid uptake) was observed. FDG-PET/CT readings not falling into any of these categories were classified as equivocal. Maximum standardized uptake value was also measured in volumes of interest and collected for each lesion.

### Outcome determination and statistical analysis

The sensitivity, specificity, positive predictive value (PPV), negative predictive value (NPV), negative and positive likelihood ratio of FDG PET/CT were determined. To compute these accuracy indices, patients were considered as having cancer if they were diagnosed with histologically proven cancer on the basis of the initial screening strategy results, or on the basis of any diagnostic test performed during the 24 months follow-up period in case of newly suspected malignancy.

We ran two sets of analysis: one considering patients with equivocal FDG-PET/CT results as positive for possible malignancy, and a second one considering patients with equivocal FDG-PET/CT results as negative for possible malignancy. For each analysis, FDG-PET/CT was considered true-positive if a cancer was diagnosed in a patient with FDG-PET/CT interpreted as positive for possible malignancy. FDG-PET/CT was considered false-positive in patients with a FDG-PET/CT interpreted as positive for possible malignancy who did not developed malignant disease during follow-up. FDG-PET/CT was considered true-negative in patients with a FDG-PET/CT interpreted as negative for malignancy who did not develop malignancy during follow-up. FDG-PET/CT was considered false-negative in patients with a FDG-PET/CT interpreted as negative for possible malignancy who developed a malignant disease during follow-up.

Statistical analysis was performed using IBM SPSS Statistics software, version 19 installed on PC. The trial is registered with ClinicalTrials.gov, number NCT00964275.

## Results

Between March 3, 2009, and August 18, 2012, 399 patients were included and randomized to one of the two study groups. Of the 200 patients allocated to FDG-PET/CT, three patients withdrew consent and refused the use of their data, two patients were found to be ineligible for the trial, and 23 patients did not present for their FDG-PET/CT or eventually refused to undergo the examination. Therefore, 172 patients were included in the present analysis. General characteristics and risk factors are presented in Table 1.

**Table 1:** General characteristics and risks factors of the population.

Characteristics	
**Age, years**	64 (48–76)
• Older than 50 years	127 (74%)
**Sex**	
• Male	94 (55%)
• Female	78 (45%)
**Venous thromboembolism**	
• Deep vein thrombosis	34 (20%)
• Pulmonary embolism ± deep vein thrombosis	138 (80%)
Tobacco use	27 (16%)
Oral contraceptives	12 (7%)
Familial history of cancer	59 (34%)
Previous malignancy	12 (7%)
Prior venous thromboembolism	49 (28%)
Alcohol intoxication	18 (10%)
Asbestos exposition	12 (7%)
Asthenia	19 (11%)
Anorexia	2 (1%)

Data are median (IQR) or n (%)

Out of the 172 patients, 10 (5.8%) patients were diagnosed with cancer at inclusion or during the 24-month follow up period. The primary site of occult malignancy included prostate in three cases, colon in two case, oropharynx in one case, lung/pleura in one case, pancreas in one case, testicle in one case and ovary/uterus in one case. Of note, malignancy was diagnosed at the initial work-up in 9 patients, and late during follow-up in one.

FDG-PET/CT was interpreted as positive for possible malignancy in 10 patients (5.8%), as equivocal in 23 patients (13.4%) and as negative in 139 patients (80.8%). Among them, malignancy was diagnosed in 7/10 (70.0%), 2/23 (8.7%) and 1/139 (0.7%) patients, respectively. Table 2 summarizes FDG-PET/CT interpretation, abnormal FDG uptake location, maximum standardized uptake value (SUV max), and final diagnosis in patients with positive or equivocal results.

**Table 2:** FDG-PET/CT interpretation, abnormal FDG uptake location, SUV max, and final diagnosis in patients with positive or equivocal results.

No.	Increased uptake location	SUV_max_	PET interpretation	Final diagnosis
1	Colon	5.3	Positive	Colon adenocarcinoma
2	Lung—Pleura	2–3.9	Positive	Lung adenocarcinoma/ pleura metastasis
3	Oropharynx	10.2	Positive	Uvula cancer
4	Kidney	3.6	Positive	No malignancy
5	Ovary/Uterus—Peritoneum	43.8–62.1	Positive	Ovary/Uterus cancer
6	Prostate	5.6	Positive	Prostate adenocarcinoma
7	Pancreas	4.6	Positive	Pancreas cancer
8	Uterus	28.6	Positive	Menstruation
9	Testicle	9.4	Positive	Testicular seminoma
10	Colon	6.7	Positive	Diverticulosis and Hyperplastic polyp
11	Parotid	4.6	Equivocal	No malignancy
12	Thyroid	2.2	Equivocal	Benign thyroid nodule
13	Larynx	2.2	Equivocal	No malignancy
14	Colon	5.9	Equivocal	Diverticulosis and Hyperplastic polyp
15	Lung	2	Equivocal	No malignancy
16	Colon	4.7	Equivocal	No malignancy
17	Oropharynx-Cervical nodes	12.4–5.9	Equivocal	No malignancy
18	Uterus	4.4	Equivocal	No malignancy
19	Larynx	3.6	Equivocal	No malignancy
20	Lung	1.9	Equivocal	No malignancy
21	Colon	7	Equivocal	No malignancy
22	Colon	3.4	Equivocal	No malignancy
23	Thyroid	2.5	Equivocal	No malignancy
24	Mediastinum—Anal canal	8.7–7.1	Equivocal	No malignancy
25	Colon—Lung—Prostate	6.1–3–3.7	Equivocal	Prostate adenocarcinoma
26	Colon	4.9	Equivocal	No malignancy
27	Gallbladder	11.4	Equivocal	Lithiasis without complication
28	Thyroid	3.6	Equivocal	No malignancy
29	Lung—Stomach	3.8–4.8	Equivocal	Prostate adenocarcinoma
30	Nasopharynx—Muscle	9.2–5.1	Equivocal	No malignancy
31	Stomach—Colon	3.9–3.9	Equivocal	No malignancy
32	Adrenal Gland	6.3	Equivocal	No malignancy
33	Duodenum—Anal Canal	8–6.7	Equivocal	No malignancy

Accuracy indices are summarized in Table 3. Grouping positive and equivocal results, the sensitivity and specificity of the FDG-PET/CT were 90% (95%Confidence Interval (CI), 60 to 98) and 85% (95%CI, 79 to 90), respectively. Grouping negative and equivocal results, sensitivity and specificity were as follows: 70% (95%CI, 40 to 89) and 98% (95%CI, 95 to 99).

**Table 3:** Accuracy of FDG-PET/CT for the diagnosis of cancer.

Accuracy indices	Positive or Equivocal vs. Negative FDG-PET/CT	Positive vs. Negative or Equivocal FDG-PET/CT
Sensitivity, % (95%CI)	90 (60 to 98)	70 (40 to 89)
Specificity, % (95%CI)	85 (79 to 90)	98 (95 to 99)
Positive predictive value, % (95%CI)	27 (5 to 44)	70 (40 to 89)
Negative predictive value, % (95%CI)	99 (96 to 100)	98 (95 to 99)
Positive likelihood ratio (95%CI)	6.1 (4.0 to 9.3)	37.8 (11.5 to 124.5)
Negative likelihood ratio (95%CI)	0.1 (0.0 to 0.8)	0.3 (0.1 to 0.8)

## Discussion

In this study we found the FDG-PET/CT to have a good accuracy for cancer diagnosis in patients with unprovoked venous thromboembolism. Sensitivity ranged from 70 to 90%, and specificity from 85 to 98% according to how equivocal test results were considered. In this work, sensitivity determines the ability of FDG PET/CT to diagnose occult cancer, i.e. a positive FDG PET/CT result when patients have malignancy. Specificity of FDG PET/CT determines the ability of the test to limit false positives examinations and so the proportion of patients who are likely to undergo unnecessary/invasives additional tests.

As with any diagnostic test, the optimal trade-off between sensitivity and specificity is not easy to determine. On one hand, considering as positive patients with a FDG-PET/CT interpreted as positive or equivocal for possible malignancy yielded a higher sensitivity (90%), and negative predictive value (99%): only one out of 139 negative patients was diagnosed with cancer during follow-up. However, the positive predictive value was low at 27%, meaning that almost three quarters of patients with a ‘positive’ FDG-PET/CT did not eventually have cancer. On the other hand, restricting positivity only to patients deemed as having a positive FDG-PET/CT for possible malignancy resulted in a higher specificity (98%) and positive predictive value (70%). This would allow limiting the proportion of patients undergoing unnecessary investigations following a positive test, at the expense of a lower sensitivity of 70%: three out of the 10 patients diagnosed with cancer in the study did not have a positive FDG-PET/CT. However, it is noteworthy that among these three patients, one was diagnosed with a colon cancer 18 months after inclusion, although his FDG-PET/CT at inclusion was clearly negative (which was confirmed by post-hoc repeated interpretation); another patient was diagnosed with a prostate cancer (PSA 6.2) although his FDG-PET/CT was equivocal at the level of the lung and the stomach; the last patient was also diagnosed with prostate cancer, his FDG-PET/CT was equivocal in the prostatic bed and his PSA level was 8.1. We would therefore argue that restricting positivity to FDG-PET/CT findings clearly positive for possible malignancy would be sufficient from a cancer screening perspective, while requiring less unnecessary additional investigations than when using a broader definition of a positive FDG-PET/CT.

Some aspects of our analysis deserve further comments. First, interpretation is difficult to standardize, and the threshold for positivity is not clearly defined. Our definition of a positive FDG-PET/CT is in line with previous studies on this topic [15–17]. However, such a definition led to highly variable proportions of positive FDG-PET/CT across studies, from 31% to 63%. The positive predictive value of the FDG-PET/CT, interpreted using this definition, differed significantly across studies, from 4% to 54%. Discrepancies between studies could be explained by small sample size, difference in the categorization of patients with ‘suspicious’ (vs. positive) findings, and different patient populations, with a prevalence of cancer ranging from 2.5 to 24%. Second, the overall incidence of cancer in our study was lower (around 6%) than anticipated: previous studies reported a 10% incidence of cancer in the year following an unprovoked venous thromboembolism [4]. The identification of predictors of cancer diagnosis in patients with venous thromboembolism could enable a better risk stratification and selection of patients requiring testing, which would lead to an improved positive predictive value of the FDG-PET/CT.

Our study has strengths and limitations. Our sample size was somewhat limited, resulting in wide confidence intervals around estimated accuracy indices. However, our data represent the largest population of venous thromboembolism patients investigated using a FDG-PET/CT. Moreover, our patient population was included in a multicenter, prospective, randomized trial, with a 24-month follow-up through outpatient clinic visits every six months. FDG-PET/CT images were centrally reviewed at the main study site by a board-certified nuclear medicine physician, blinded from patients’ history, initial FDG-PET/CT interpretation, and follow-up status but without specific reproducible interpretation criteria for a positive FDG-PET/CT. However, an interobserver agreement was not performed in this study which could limit the robustness of the results. A possible way to improve the diagnostic performance of FDG-PET/CT in the setting of cancer screening in patients with unprovoked venous thromboembolism would be to define specific interpretation criteria dedicated to this clinical setting, in order to minimize findings of low clinical relevance. FDG-PET/CT interpretation criteria have been well defined in specific settings (e.g. tumor staging, restaging or interim therapeutic evaluation) [1,2]. However, defining criteria for screening might be particularly challenging for whole-body imaging, given that patients with unprovoked venous thromboembolism may develop any cancer type, location or stage.

In conclusion, FDG-PET/CT showed a good accuracy for the screening of occult cancer in patients with unprovoked venous thromboembolism. Remaining challenges include the need to define specific and standardized interpretation criteria dedicated to this clinical indication, and the need to better select high-risk patients who would get the most benefit from screening with FDG-PET/CT.
